# Berry phase mechanism of the anomalous Hall effect in a disordered two-dimensional magnetic semiconductor structure

**DOI:** 10.1038/srep17158

**Published:** 2015-11-24

**Authors:** L. N. Oveshnikov, V. A. Kulbachinskii, A. B. Davydov, B. A. Aronzon, I. V. Rozhansky, N. S. Averkiev, K. I. Kugel, V. Tripathi

**Affiliations:** 1 National Research Center Kurchatov Institute, Moscow 123182, Russia; 2 Low Temperature Physics Department, M.V. Lomonosov Moscow State University, Moscow 119991, Russia; 3 P.N. Lebedev Physical Institute, Russian Acad. Sci., Moscow 119991, Russia; 4 Ioffe Institute, Russian Acad. Sci., St. Petersburg 194021, Russia; 5 Peter the Great St. Petersburg Polytechnic University, St. Petersburg 195251, Russia; 6 Institute for Theoretical and Applied Electrodynamics, Russian Acad. Sci., Moscow 125412, Russia; 7 Materials Science Division, Argonne National Laboratory, Lemont, IL 60439, USA; 8 Department of Theoretical Physics, Tata Institute of Fundamental Research, Mumbai 400005, India

## Abstract

The anomalous Hall effect (AHE) arises from the interplay of spin-orbit interactions and ferromagnetic order and is a potentially useful probe of electron spin polarization, especially in nanoscale systems where direct measurement is not feasible. While AHE is rather well-understood in metallic ferromagnets, much less is known about the relevance of different physical mechanisms governing AHE in insulators. As ferromagnetic insulators, but not metals, lend themselves to gate-control of electron spin polarization, understanding AHE in the insulating state is valuable from the point of view of spintronic applications. Among the mechanisms proposed in the literature for AHE in insulators, the one related to a geometric (Berry) phase effect has been elusive in past studies. The recent discovery of quantized AHE in magnetically doped topological insulators - essentially a Berry phase effect - provides strong additional motivation to undertake more careful search for geometric phase effects in AHE in the magnetic semiconductors. Here we report our experiments on the temperature and magnetic field dependences of AHE in insulating, strongly-disordered two-dimensional Mn delta-doped semiconductor heterostructures in the hopping regime. In particular, it is shown that at sufficiently low temperatures, the mechanism of AHE related to the Berry phase is favoured.

The anomalous Hall effect (AHE) is widely observed in ferromagnets in the presence of spin-orbit interaction^1^. It accurately indicates the onset of spin polarization of the carriers – a key requirement for spintronic devices. The need for electrostatic gate-control of spin-polarization in typically planar device geometries requires an understanding of AHE in two-dimensional (2D) ferromagnetic semiconductors in the insulating state. Metallic structures are not as useful as screening by mobile charge carriers makes gating difficult. Existing theories of AHE in semiconductors are based on the consideration of different spin-dependent scattering processes^2^,^3^,^4^, or geometric (Berry–Pancharatnam) phase effects^5^,^6^, and have been quite successful in describing AHE in the metallic regime (such as in Mn-doped GaAs). Much less understood is AHE in the insulating, strongly-disordered hopping regime, and while there are some proposals^7^,^8^ in the literature, experiments^12^ have not been able to resolve important questions such as the dominant mechanisms governing AHE in different parameter regimes. In particular, an intriguing mechanism of AHE proposed for disordered ferromagnetic insulators^8^, involving a subtle quantum interference of triads of misaligned spin states on the dominant conducting paths, has proved very difficult to confirm. Such a purely geometrical quantum interference effect is an instance of the well-known Berry-Pancharatnam phase. While the Berry phase mechanism of AHE has eluded detection in ferromagnetic semiconductors in the insulating state, it has seen spectacular confirmation in other insulating systems with simultaneous presence of ferromagnetic polarization and strong spin-orbit coupling. The one closest to our case concerns the recent discovery^9^ of quantized AHE on 2D surfaces of 3D topological insulators in the presence of a small concentration of magnetic impurities that undergo ferromagnetic ordering. The quantized AHE is the result of two-dimensionality and strong spin-orbit coupling in the topological insulator and is directly related to the quantization of the geometric flux accumulated by Bloch states around the Brillouin zone, analogous to the quantized ordinary Hall effect^10^. Likewise, a novel thermal Hall effect recently reported^11^ in insulating bosonic 2D spin liquids is in fact an AHE associated with geometric phase effects. Motivated thus, we have performed a detailed experimental study of AHE in strongly-disordered insulating 2D semiconductor heterostructures consisting of a quantum well spatially separated from a layer of Mn-dopants that is the source of ferromagnetism. Our main finding concerns strong evidence of the geometrical phase related mechanism for AHE at sufficiently low temperatures. We also find that at higher temperatures, AHE is governed by an alternative mechanism based on a spin-dependent hopping of 2D holes in a quantum well (QW) interacting with remote magnetic layer, which has been predicted^7^ and observed^12^ for bulk magnetic semiconductors.

## Methods

**Figure 1:**
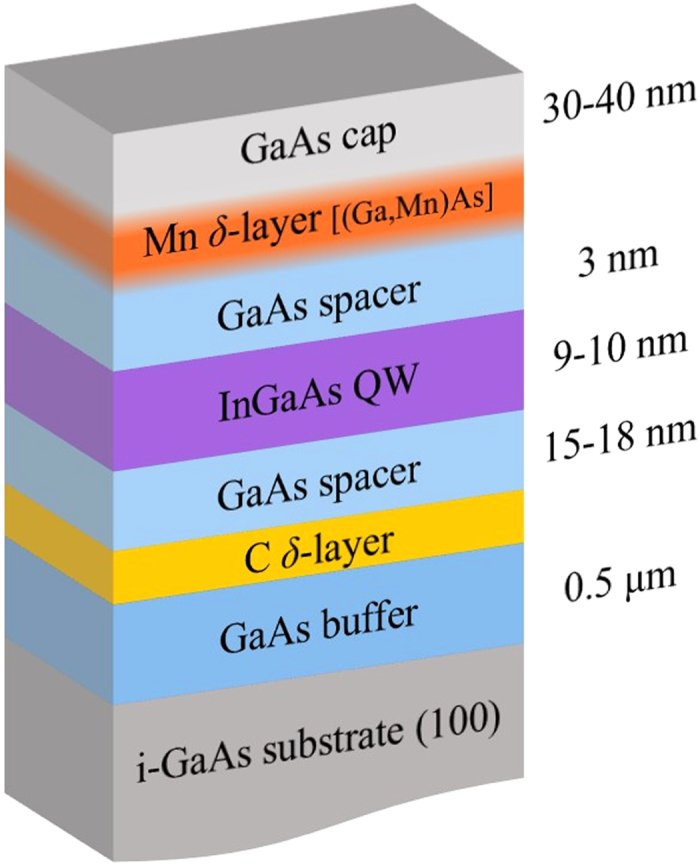
Schematic layout of the two-dimensional magnetic semiconductor heterostructures used in the study. Resistivity and Hall measurements are performed for the InGaAs quantum well (QW) layer. The Mn *δ*–doping, which in reality is a (Ga, Mn)As layer (see text) due to diffusion of Mn atoms in the GaAs host, is a source of charge carriers as well as ferromagnetism. The intensity of shading of the Mn layer indicates the inhomogeneity of the doping due to diffusion of Mn atoms. The carbon *δ*–layer provides additional holes to make the GaAs layer a p-type semiconductor, while the buffer layer is intrinsic GaAs. The heterostructure design provides a favourable platform for gate control of ferromagnetic properties.

GaAs/*δ*–Mn/GaAs/In_*x*_Ga_1−*x*_As quantum well (QW) structures were grown by metal-organic chemical vapour deposition (MOCVD) methods on semi-insulating GaAs substrates, using laser deposition of Mn. Two of the samples, M1 and M2, were cut from the same wafer. The main part of each sample is the In_*x*_Ga_1−*x*_As QW sandwiched between two GaAs layers. One of them acts as a 3 nm spacer separating the QW from the Mn *δ*-layer with the effective Mn concentration of 0.3 monolayer. Figure 1 shows a schematic of the sample structure. Our previous X-ray studies^13^ have shown that the composition of this layer is indeed Ga_1−*x*_Mn_*x*_As with Mn distributed over a range of 2 nm, and around 5% Mn-doping at the centre of the layer giving a QW–Mn separation of 2–2.5 nm. Bulk GaAs films with such a concentration of Mn are usually ferromagnetic with the Curie temperature slightly above 100 K. Mn in GaAs and in our structures plays a dual role as an acceptor and a magnetic impurity. Indium content *x* in the strained channel layer In_*x*_Ga_1−*x*_As is 0.216 for both samples (see an earlier paper by some of us^14^ for a detailed description of the sample structure). The magnetic field and temperature dependences of longitudinal and Hall resistivities were analyzed in the temperature range 2–80 K, and for magnetic fields up to 8 T. We find that the hole concentration *p* obtained from the Hall measurements is almost temperature independent below 35–40 K (with *p* ≈ 0.4 × 10^12^ cm^−2^). The spatial distribution of Mn atoms within the impurity layer is inhomogeneous^15^, which creates fluctuation potentials and phase separation in the impurity (Mn) layer as well as in the transport channel (QW)^13^. The parameters of two studied samples M1 and M2 are slightly different. In particular, the hole densities in M1 and M2 (in units of 10^12^ cm^−2^) are, respectively, 0.31 and 0.36 at 4.6 K; 0.33 and 0.39 at 32 K. The corresponding mobilities (in units of cm^2^/Vs) are, 350 and 371 at 4.6 K; 850 and 775 at 32 K. This affects the fluctuation potential and leads to some difference in the low temperature features of these samples. Parameters of the fluctuation potential and its effect on the heterostructure conductivity and magnetic properties have been discussed^16^,^17^ earlier.

**Figure 2:**
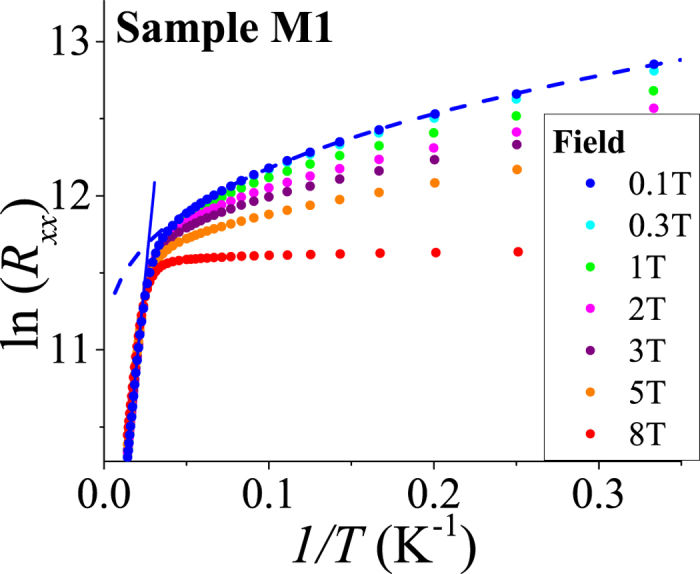
Temperature dependence of the longitudinal resistance for sample M1 for different values of perpendicular magnetic field *B*. The Arrhenius law at higher temperatures (straight line fit) gives way to a 2D Mott variable range hopping law at lower temperatures (dashed line fit).

Figure 2 shows the temperature dependence of the longitudinal resistivity for sample M1 for different values of perpendicular magnetic field *B*. A transition from activated to variable-range hopping (VRH) conduction is visible as the temperature is lowered. In the hopping regime, a better fit is achieved by the 2D Mott VRH law ln *R*_*xx*_ ~ (1/*T*)^1/3^ compared to the Efros–Shklovskii VRH ln *R*_*xx*_ ~ (1/*T*)^1/2^ signifying that long-range Coulomb interactions are not important in this temperature range. From the intersection of Arrhenius and VRH fitting curves, we estimate the temperature *T*_hop_ at which the transition from drift to hopping transport occurs. For sample M1, we find *T*_hop_ = 37 K (*B* = 0.1 T) and 38.5 K (*B* = 8 T).

**Figure 3:**
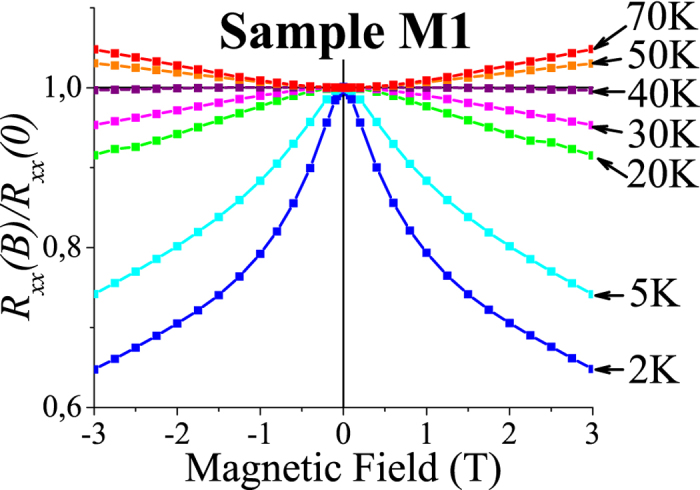
Magnetic field dependence of *R*_*xx*_(*B*)/*R*_*xx*_(0) at different temperatures. As the temperature is decreased, the curvature changes sign around *T* = 40 K. It is argued in the text that the negative sign at low temperatures is mainly not a quantum interference effect but rather a sign of increasing ferromagnetic correlations.

Figure 3 shows the results of magnetoresistance measurements. Upon lowering the temperature, a transition from positive to negative magnetoresistance occurs around *T* = 40 K, which incidentally is very close to *T*_hop_. We attribute the positive magnetoresistance to the localizing (orbital) effect of the magnetic field in the drift regime. In the hopping regime, an opposing contribution to the magnetoresistance comes from the dependence of the hopping rate on the alignment of the magnetic moments. We thus believe that the negative magnetoresistance is related to spin-dependent tunnelling and is an indication of the trend to onset of ferromagnetic order rather than a quantum interference effect^16^,^18^.

The Mn impurity layer determines the magnetic properties. Exchange interaction mediated by itinerant carriers in the (Ga, Mn)As layer^19^ and free carriers in the QW^20^,^21^ lead to ferromagnetic (FM) ordering of the Mn magnetic moments. The Mn-layer is phase separated into ferromagnetic regions (with a high Mn content) inside a non-ferromagnetic matrix, and undergoes a percolation transition to a long-range ferromagnetic state at low temperatures. In these 2D disordered magnets (unlike 3D counterparts), the Curie temperature, at which local ferromagnetic order appears (as detected through AHE measurements) and the temperature *T*_*c*_, at which ferromagnetic clusters begin percolating (visible as a peak in the temperature dependence of *dR*_*xx*_/*dT*) can be rather far apart^16^. The structure demonstrates ferromagnetic ordering^15^,^16^ with *T*_*c*_ = 24 *K* estimated from the peak in *dR*_*xx*_(*T*)/*dT* vs *T*^16^,^22^.

**Figure 4:**
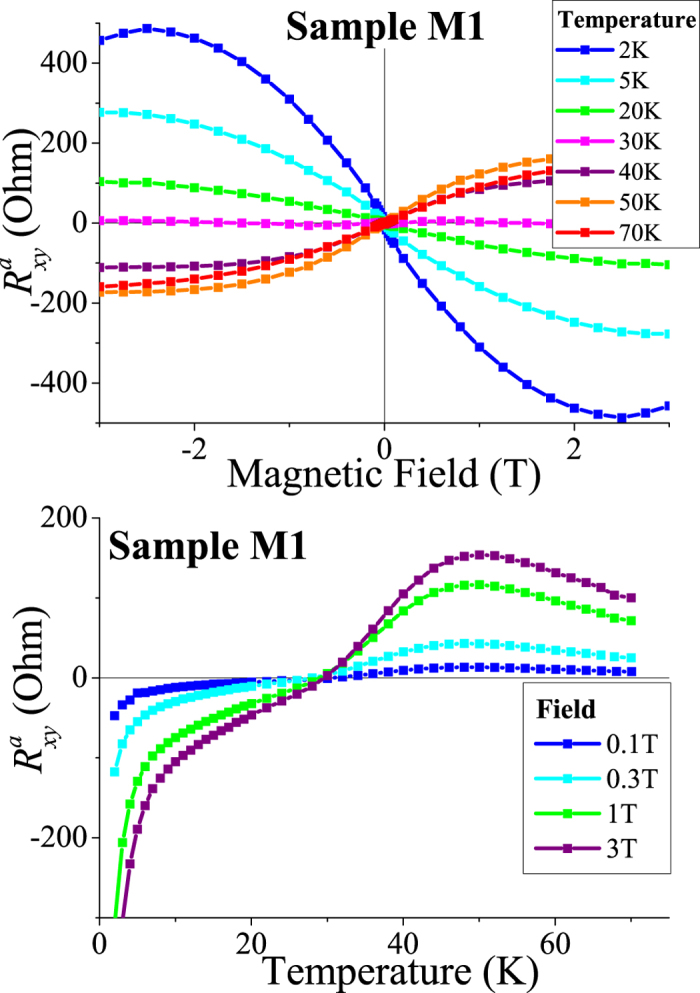
Magnetic field and temperature dependences of the anomalous Hall resistance 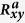 of sample M1. Saturation is observed above fields of around 2 T. The anomalous Hall resistance increases with temperature over a considerable range - a surprising feature also observed in two-dimensional thin films of GaMnAs^23^. 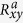 also changes sign around *T*_*c*_.

The AHE contribution is obtained from the full Hall signal by separating the linear-in-*B* part, which is possible if the sample magnetization saturates. In Fig. 4, we show thus obtained magnetic field and temperature dependences of the anomalous Hall resistivity 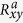 we have obtained in Fig. 4. The field dependences typically tend to saturate above 2 T (see also refs 13,15,16) with a small downturn in 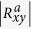, most pronounced in the curve corresponding to the lowest temperature. In our opinion, the non-monotonic behavior at *B* > 2 T is due to the onset of a strong magnetic field regime, where the magnetic length becomes comparable to the distance between magnetic impurities. However, as the downturn is more evident at lower temperatures, we cannot completely rule out the possibility that it could be a trace of the Berry phase mechanism described below. One striking feature of the observed AHE is the increase of 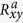 with temperature, very similar to the earlier observation^23^ in thin GaMnAs layers. Reduced dimensionality, a common feature of the two systems, may be the cause of such anomalous behaviour. Note also that in our structures, 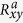 changes sign upon cooling around *T*_*c*_, the magnetic percolation temperature.

## Results and Discussion

**Figure 5:**
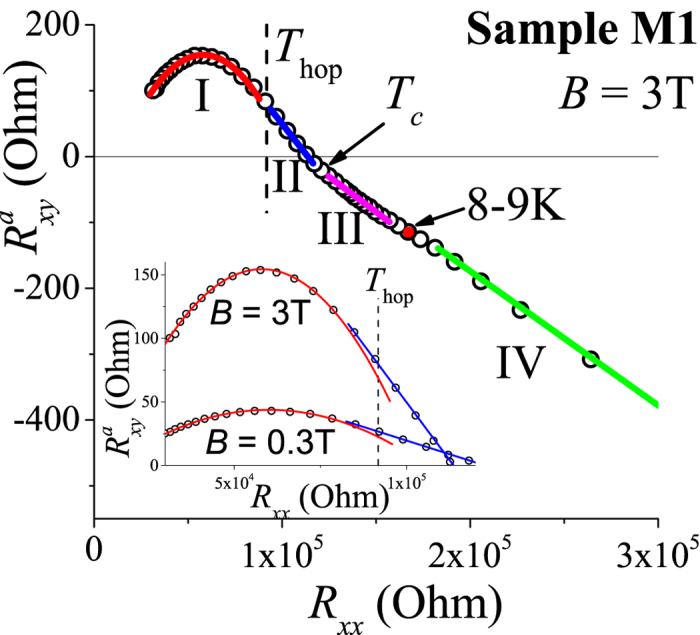
Parametric dependence of anomalous Hall resistivity 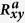 on longitudinal resistivity *R*_*xx*_ for *B* = 3 T fitted by a linear function at low temperatures and by a quadratic function at higher temperatures. Inset shows linear-to-quadratic behaviour change around *T*_hop_.

Different physical mechanisms of AHE may become relevant as the parameters are varied. In bulk metals, the scaling of 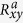 with longitudinal resistivity is commonly used to determine the dominant physical mechanism. A linear dependence 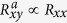 is a signature of the scattering-dependent skew-symmetric mechanism, while a quadratic dependence 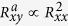 indicates the side-jump or intrinsic mechanisms, the latter being scattering-independent. The hopping transport regime needs a substantially different theoretical approach. One possible mechanism of AHE in a 3D disordered ferromagnet in the hopping regime has been suggested^7^ based on spin-dependent hopping between the sites as a consequence of spin-orbit interaction and spin polarization of the carriers in effective exchange mean field. The theory predicts a linear parametric dependence of 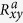 on *R*_*xx*_,


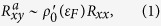


where *ρ*_0_ is the density of states at the Fermi level. The sign of AHE relative to the ordinary Hall effect may vary depending on the position of the Fermi level within the impurity band. An experimental observation of both signs of AHE in (Ga, Mn)As hopping transport was reported and interpreted using this theory^12^. A different model for the AHE in the hopping regime was developed for manganites^8^, where AHE arises from a Berry–Pancharatnam geometric phase due to misalignment of local magnetic moment axes and thus vanishes as the magnetization saturates. It was claimed^12^ that the geometric phase contribution is not relevant in magnetic semiconductors on the basis of their observation that AHE does not vanish at saturation magnetization. This argument however is correct only if a single mechanism dictates AHE. Below we make a careful analysis of the field dependence of AHE and argue that in our experiments we observe both hopping AHE mechanisms mentioned above along with the AHE reminiscent of side jump or intrinsic mechanisms in the band conductivity regime. Figure 5 shows anomalous Hall data for sample M1 at *B* = 3 T. We observed four temperature ranges with different parametric dependences *R*_*xy*_(*R*_*xx*_) corresponding to (with decreasing temperature) the crossover to hopping transport at *T*_hop_, ferromagnetic ordering at *T*_*c*_, and to the puzzling appearance of a new AHE mechanism at low temperature which we describe below. At higher temperatures, where conductivity is determined by drift transport of holes at the percolation level, we find the sign of AHE is positive relative to the ordinary Hall effect and is described by a quadratic fit, 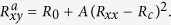 This is reminiscent of scattering-independent mechanisms seen^1^ in metallic (Ga, Mn)As. A linear relation 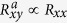 provides a good fit to the data below the hopping transition temperature *T*_hop_. Here, we observe a decrease of 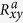 with lowering *T* (and rising *R*_*xx*_). At *T*_*c*_ ≈ 24 K, corresponding to the magnetic percolation transition, the slope 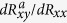 changes together with the sign of 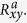 The low-temperature regime in Fig. 5 has another peculiarity - an inflection point corresponding to a temperature of 8–9 K. At this point, the second derivative becomes zero, while the first derivative has a peak. We argue that in this lowest temperature range the AHE is governed by the Berry–Pancharatnam geometric phase mechanism for the first time observed in two-dimensional system.

**Figure 6:**
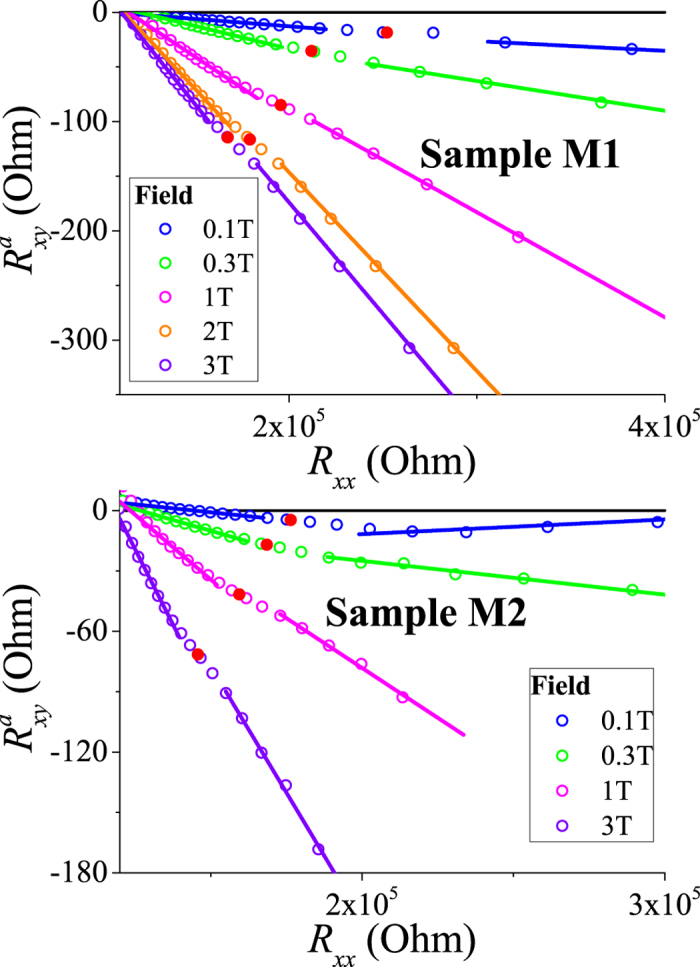
Parametric dependences (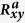 vs. *R*_*xx*_) at low temperatures for different values of the magnetic field. The behaviour of 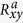 below the inflection points (shown as red dots) on the curves shows strong sensitivity to magnetic fields as small as 0.1 T, and this effect goes away at higher fields ~3 T, where the impurity magnetization is saturated. This contribution is opposite in sign compared to the high field trend and even manifests as an upturn in sample M2. An interpretation in terms of a geometric phase effect is given in the text.

First, let us summarise the emerging physical picture for AHE in our system in the temperature ranges I–III. We have two contributions to conductivity: (a) hopping between metallic droplets and (b) drift conductivity inside them and at percolation level. At high temperatures (Region I), drift conductivity prevails, AHE is positive and a quadratic parametric dependence of 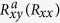 fits experimental results (see the lower inset of Fig. 5). In this region the AHE is governed by the mechanism, reminiscent of the side-jump or dissipationless intrinsic ones, while the current is carried by the delocalized band states. With rising magnetic field, the magnetization increases resulting in enhancement of AHE. Now let us discuss AHE in the hopping regime. It is instructive to recall the mechanism of the ordinary Hall effect in the hopping regime. As was first shown by Holstein^24^, the Hall conductivity *σ*_*xy*_ in disordered insulators is determined by the Aharanov–Bohm flux Φ = ∫_Δ_**A** ⋅ *d***l** threading elementary triangles on the conducting backbone:


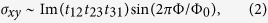


where *t*_*ij*_ denote the hopping matrix elements between sites *i* and *j*, and in our case metallic droplets inside the QW. The presence of spin-orbit interaction makes hopping spin-dependent, and together with the spin polarization of the holes in a exchange mean field of Mn ions, one gets AHE exactly as described in^7^. In Region II between *T*_hop_ and *T*_*c*_, hopping transport starts to dominate over drift band conductivity. We assume in our case the hopping contribution to AHE is of the opposite sign to the band conductivity contribution. In the region II, however, while the hopping conductivity dominates over drift the drift contribution to AHE is still stronger and 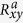 maintains a positive sign. Physically, this is possible as hopping contributes less to the Hall effect than it does to the conductivity. With further decreasing temperature, the drift contribution to AHE is eventually superseded by the hopping one. The sudden enhancement of spin-dependent tunnelling at the magnetic percolation temperature *T*_*c*_ strongly enhances the hopping contribution and causes a sign change in 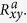 Further, since the establishment of long-range ferromagnetic order weakens the temperature dependence of magnetization (and 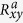), the slope of 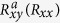 diminishes upon crossing *T*_*c*_. Figure 6 shows the evolution of parametric dependences for both samples in different magnetic fields. Since the drift and spin-dependent hopping contributions increase with magnetic field (through its effect on the magnetization) in the entire temperature range, the gradient of 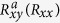 is expected to increase with *B*. However, in Region III below *T*_*c*_, the dependence of the gradient of 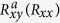 on magnetic field grows slower as it should be in accordance with the weaker temperature dependence of magnetization.

Finally, we discuss AHE at very low temperatures, below the temperature of the inflection point (Region IV). The different roles played by the spin-orbit interaction (SOI) in the hopping conductivity need to be appreciated here. First, it generates AHE by imparting a spin-dependence to the hopping of the spin-split carriers between localized sites in the QW. This contribution exists over the entire temperature range provided there is a spin polarization of the carriers due to the exchange mean field. This contribution is normally regarded as the main cause for AHE in the hopping regime discussed in the existing literature for bulk GaMnAs^7^,^12^,^25^. We also believe this mechanism to be of key importance for the temperature regions II and III. The second role of SOI is that it generates a Dzyaloshinskii–Moriya (DM) interaction of magnetic moments on a triad. In this case as a carrier hops around this triangle, it picks an additional geometric (Berry–Pancharatnam) phase Ω/2, which is the quantum phase associated with the wavefunction overlaps 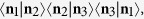 tan(Ω/2) = (**n**_1_ ⋅ **n**_2_ × **n**_3_)/(1 + **n**_1_ ⋅ **n**_2_ + **n**_2_ ⋅ **n**_3_ + **n**_3_ ⋅ **n**_1_). This geometric phase, essentially the solid angle defined by the three unit vectors **n**_*i*_ takes the place of the Aharanov–Bohm phase in the ordinary Hall effect, so that


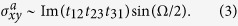


A subtlety that needs commenting on is that upon averaging over the spin orientations, Ω vanishes since reflection of the vectors about any plane containing the direction of magnetization (keeping the magnetization fixed) reverses the sign of **n**_1_ ⋅ **n**_2_ × **n**_3_. However a finite spin-orbit interaction lifts the degeneracy (see e.g.^8^,^26^) by introducing the DM interaction of spins in the triad, and this results in a finite anomalous Hall contribution proportional to *sin*(Ω/2). The temperature then needs to be smaller than the DM scale to prevent mixing of these states. This mechanism does not require spin splitting of charge carrier energies; moreover, it does not exist in a strong exchange mean field as it needs the on-site magnetic moments to be partly disoriented. This contribution is small near *T*_*c*_ where the magnetization is small and steadily increases on cooling, being peaked at magnetization of about 0.35 of the saturation value^8^. Thus the geometric phase contribution exists only for temperatures below the inflection point (Region IV) and at low magnetic fields. Because the above two contributions to AHE are physically different, they can be of different sign. If we assume that, then the AHE and parametric dependence in Region IV (and others) is easily understood. Since the DM geometric contribution is suppressed by a magnetic field, we observe in Fig. 6 that the upturn in the AHE seen in Region IV, reverts to the trend in Region III at *B* = 3 T. The same behaviour can also be seen even more clearly in sample M2. The difference in the behaviour of samples M1 and M2 is due to small differences in their parameters (see Sec. Methods) arising from the spatial inhomogeneity in Mn doping.

## Conclusions

In summary, we have performed detailed measurements and analysis of the anomalous Hall resistance 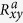 and longitudinal resistance *R*_*xx*_ of two-dimensional ferromagnetic semiconductor heterostructures that we have earlier established to have two magnetic transitions corresponding to appearance of local ferromagnetic order and a percolation transition at a lower temperature *T*_*c*_. At temperatures around the local ferromagnetic transition, the parametric scaling of 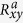 with *R*_*xx*_ is observed to be parabolic, reminiscent of side-jump or intrinsic mechanisms in bulk metallic ferromagnets. At lower temperatures, 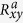 scales linearly with *R*_*xx*_, which has an insulating temperature dependence. At *T*_*c*_, the linear scaling persists and the sign of 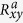 reverses. The parametric dependence of *R*_*xy*_ on *R*_*xx*_ in the hopping regime is expected to be linear^7^,^27^ since both Hall and longitudinal conductances crucially depend on the rate of phonon-assisted direct hops. This is reminiscent of scattering-dependent mechanisms of AHE in the metallic regime. The averaging over particular distribution of triads is known to be non-trivial and can bring additional corrections to the parametric dependence^28^. However, our experimental results fit well to a simple linear dependence and we are not able to discriminate between these two predictions. Therefore, additional measurements are needed and we leave this matter for future studies. At even lower temperatures (around 8–9 K), there is evidence of a new contribution to 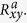 The scaling with *R*_*xx*_ continues to be linear but this contribution to 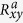 has a positive sign, and it has a strong dependence on the applied magnetic field. For saturating (and higher) magnetic fields, this contribution becomes vanishingly small. We find this behaviour to be consistent with a geometric Berry–Pancharatnam phase picture including the DM interaction. For the Berry–Pancharatnam geometric phase mechanism of AHE in the hopping regime, the theory of parametric scaling of *R*_*xy*_ with *R*_*xx*_ is not well developed. Our experimental data for the lowest temperature regime fits well to a linear dependence. To our understanding, this is the first observation of the geometric phase mechanism of the anomalous Hall effect in 2D semiconductor heterostructures. In hindsight, the 2D nature of the structure facilitated the geometric phase mechanism because here, unlike the 3D case, there is a window of temperatures above the percolation transition where one has local ferromagnetism but the moments are misaligned on longer length scales.

## Additional Information

**How to cite this article**: Oveshnikov, L. N. *et al*. Berry phase mechanism of the anomalous Hall effect in a disordered two-dimensional magnetic semiconductor structure. *Sci. Rep*. **5**, 17158; doi: 10.1038/srep17158 (2015).
